# Association of low back pain with muscle weakness, decreased mobility function, and malnutrition in older women: A cross-sectional study

**DOI:** 10.1371/journal.pone.0245879

**Published:** 2021-01-25

**Authors:** Satoshi Kato, Satoru Demura, Kazuya Shinmura, Noriaki Yokogawa, Tamon Kabata, Hidenori Matsubara, Yoshitomo Kajino, Kentaro Igarashi, Daisuke Inoue, Yuki Kurokawa, Norihiro Oku, Hiroyuki Tsuchiya

**Affiliations:** Department of Orthopaedic Surgery, Graduate School of Medical Sciences, Kanazawa University, Kanazawa, Japan; Western University, CANADA

## Abstract

**Background:**

Low back pain (LBP) and decreased mobility function are common problem among older people. Muscle weakness has been reported as a risk factor for these conditions, and exercise therapy can improve them. We created a novel exercise device that also measures abdominal trunk muscle strength. Malnutrition has also emerged as a major problem among older people. Muscle is a direct key linking decreased mobility function and malnutrition. This study aimed to examine the associations of LBP with not only decreased physical function and muscle weakness but also nutritional status of older people.

**Methods:**

We examined the associations of LBP with muscle weakness, decreased mobility function (locomotive syndrome [LS]), and malnutrition among older women. The study included 101 female patients aged 60 years or older scheduled to undergo surgery for degenerative lower extremity diseases. Preoperatively, physical tests including abdominal trunk muscle strength assessment using the device and laboratory tests were conducted. Subjects with LBP (numerical rating scale ≥2; range, 0–4) during the preceding month were allocated to the LBP group (n = 36). Other subjects were allocated to the non-LBP group (n = 65).

**Results:**

The LBP group had lower abdominal trunk and knee extensor muscle strength, lower serum albumin, and hemoglobin levels as blood biomarkers associated with malnutrition risk, and higher LS test scores than the non-LBP group. A multivariate analysis showed that abdominal trunk muscle weakness and advanced LS were associated with LBP. LBP intensity was negatively correlated with abdominal trunk and knee extensor muscle strength and positively correlated with the LS test score. The serum hemoglobin level was negatively correlated with the LS test score.

**Conclusion:**

Abdominal trunk muscle weakness and decreased mobility function were associated with LBP among older women.

## Introduction

Low back pain (LBP) is a common problem globally [1]. LBP affects approximately 80% of people at some time in life [2, 3]. The prevalence of LBP is related to aging and is the main cause of disability for older people in developing and developed countries [4, 5]. Trunk muscle weakness has been reported as a risk factor for LBP [6, 7]. In a systemic review, Hayden et al. [8] reported that among various types of exercise therapies for chronic LBP, strengthening exercises were the most effective for improving physical functions. For older patients with chronic LBP, compliance with performing necessary exercise and the motivation to exercise are generally limited [9, 10]. Unfortunately, research of exercise for chronic LBP has frequently focused on the working population and has excluded the older population due to age-related co-morbidities [10–12]. A significant number of older patients with chronic LBP are unable to continue their recommended exercise regimen because they have pain, deformities, and/or loss of flexibility in the spine and extremities and because of muscle weakness [12, 13].

Decreased mobility function is an inevitable outcome of aging, and an increasing proportion of older individuals require care because of impaired mobility [14, 15]. Because increasing health care costs associated with aging societies impose a growing economic burden, there is an urgent need to extend the healthy life expectancy and reduce health disparities. Locomotive syndrome (LS), proposed by the Japanese Orthopaedic Association, is a condition characterized by reduced mobility due to impairment of the locomotive organs [16]. The progression of this syndrome limits the ability of individuals to carry out activities of daily living. LS is associated with multiple factors, including osteoporosis, osteoarthritis, and sarcopenia, which can cause LBP [17, 18]. Exercise therapy can improve the mobility function of patients with LS. However, because a significant proportion of patients are older adults with degenerative locomotive organ diseases, careful selection of the type and intensity of exercise is necessary [18].

**Fig 1:**
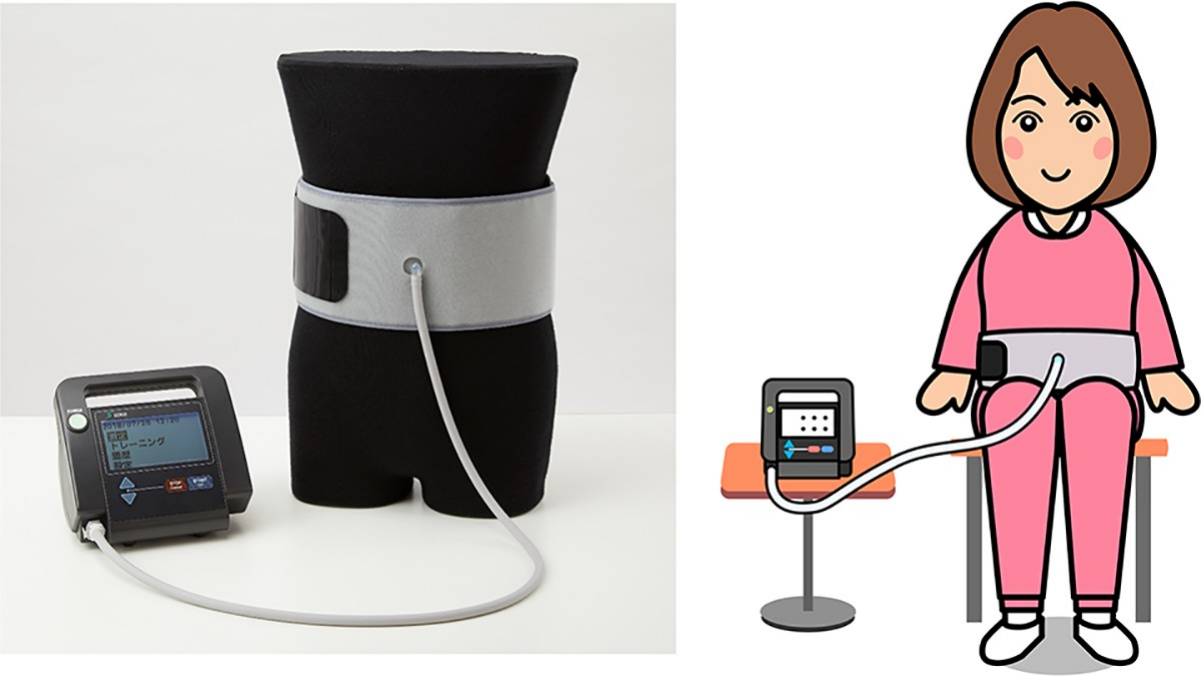
Novel exercise device for abdominal trunk muscles. (Left) Photograph of the device. (Right) Artistic rendering of a patient wearing the device.

We created a novel exercise device for abdominal trunk muscles (Fig 1) (trunk muscle exercise device; Nippon Sigmax Co., Ltd., Shinjuku-ku, Tokyo, Japan) [19]. Using this device, subjects can strengthen abdominal trunk muscles and measure their muscle strength while sitting, without moving the trunk or lumbar spine. We have previously demonstrated that this device reliably measures the strength of abdominal trunk muscles [20].

Similar to decreased mobility function, malnutrition is a highly prevalent condition among older people and is linked to higher morbidity, higher mortality, and lower quality of life [21, 22]. Malnutrition can also contribute to musculoskeletal health and is associated with chronic musculoskeletal pain among older adults [23, 24]. There are different mechanisms in the development of malnutrition in older adults and others. Chronic musculoskeletal pain including LBP and decreased physical function reduce the mobility of the elderly. Immobility attributable to musculoskeletal conditions is associated with sarcopenia and decreased oral food intake [25]. Decreased mobility function and malnutrition are two conditions that are prevalent in older people. These two conditions may be correlated and may potentiate each other, resulting in poor health-related outcomes [21, 26]. Muscle is a direct key linking decreased mobility function and malnutrition. Decreased muscle strength has been reported as an indicator of decreased mobility function [27], and decreased muscle volume has been presented as an outcome of malnutrition [28].

A previous study involving a small sample size demonstrated the links among LBP, muscle strength, and decreased mobility function of adults during the seventh and eighth decades of life [29]. To date, the association of LBP with muscle strength-related physical function and nutritional status has not been well-examined; however, it is an important issue worth considering in more detail. In the present study, we therefore investigated the associations of LBP with muscle strength, mobility function, and nutritional status among more than 100 older women.

## Materials and methods

### Ethics statement

This study was approved by the Kanazawa University ethics committee (ethic approval code: 2015–063), and written informed consent was obtained from each subject.

### Subjects

Between January 2016 and December 2017, we recruited 119 consecutive patients 60 years or older who were slated to undergo surgery because of degenerative lower extremity diseases at Kanazawa University hospital and who agreed to participate in preoperative physical tests. Male patients were excluded from the analysis because there were only 13 which was not sufficient, and because the analyses of men and women needed to be separate owing to the large differences in muscle strength between men and women. Patients who had previously undergone spine surgery or had been diagnosed with rheumatological diseases were excluded from the study. Patients whose knee extensor muscle strength could not be assessed adequately owing to pain from degenerative diseases of the lower extremities and patients whose physical or laboratory data were missing were also excluded from the analysis. Finally, a total of 101 female patients 60 years or older were evaluated duringthe study (Table 1).

**Table 1:** Patient characteristics and inclusion/exclusion criteria of the study.

**Characteristic**	
**No. of subjects**	101
**Age (years), mean ± SD [range]**	69.7 ± 6.4 [60–86]
**Height (cm), mean ± SD [range]**	151.3 ± 6.1 [129.8–165.4]
**Weight (kg), mean ± SD [range]**	54.1 ± 8.8 [35.0–81.4]
**BMI (kg/cm**^**2**^**), mean ± SD [range]**	23.7 ± 3.8 [16.7–36.7]
**Grip power (kg), mean ± SD [range]**	20.7 ± 5.7 [8.0–39.5]
**Degenerative diseases of the lower extremities treated with surgeries**	Hip joint diseases (68)
	Knee joint diseases (15)
	Foot & Ankle joint diseases (18)
**Inclusion criteria of the study**	
• **60 years-old or older female**	
• **Capable of understanding the content of the study, and giving the informed consent**	
• **Capable of performing preoperative physical tests without any difficulties in this study**	
• **Capable of obtaining preoperative laboratory data in this study**	
**Exclusion criteria of the study**	
• **Male or younger than 60 years**	
• **History of spinal surgery**	
• **Comorbidity of rheumatological diseases**	
• **Missing data in the physical or laboratory tests**	

BMI, body mass index; SD, standard deviation

### Exercise device

The exercise device used during this study has a design similar to that of a sphygmomanometer, with an inflatable cuff and a mechanical manometer to measure pressure (Fig 1). The procedure for measuring abdominal trunk muscle strength (ATMS) of the study participants has been described in detail elsewhere [19, 20]. When measuring strength or performing strengthening exercises using the device, subjects were able to contract the abdominal trunk muscles against the pressure from the inflated cuff while sitting, without the need to move the trunk. The isometric muscle contraction does not strain the spine or extremities. Previous studies have reported that no participants experienced adverse events, including exacerbation of back pain or onset of pain or discomfort in the abdomen, during exercise or strength assessment using the device [19, 20, 29, 30].

### Assessment

We measured the body height, body weight, and body mass index (BMI) of the participants. Grip power was measured using a dynamometer (TTM Dynamometer; Tsutsumi, Tokyo, Japan). Knee extensor muscle strength (KEMS) was measured using a hand-held dynamometer (μTas F-1; ANIMA Corp., Tokyo, Japan). The KEMS values were divided by body weight (N/kg). To measure KEMS, subjects were seated in an elevated chair with the knees flexed 90°, feet above the floor, and arms crossed in front of the body. The dynamometer was placed on the anterior surface of the leg, 10 cm proximal to the malleoli. Subjects were instructed to push against the dynamometer by attempting to straighten the knees [31]. The right and left grip power and KEMS were measured once, and the higher strength values of each were recorded. We measured the ATMS twice using the device and recorded the higher measurement. We obtained responses to the 25-Question Geriatric Locomotive Function Scale (GLFS-25), which is a tool used to assess LS [32]. A high GLFS-25 score indicated advanced LS (decreased mobility function). We also obtained each patient’s 5-point numerical rating scale (NRS) score for the back, LBP, or buttocks (0 = no pain to 4 = severe pain) from the response to question 2 of the GLFS-25. Subjects with LBP (NRS score ≥2; range 0–4) during the past 1 month were allocated to the LBP group (n = 36). The other subjects were allocated to the non-LBP group (n = 65). Decreased mobility function (i.e., LS) was assessed using the total score of 24 questions (the GLFS-24) of the GLFS-25 after eliminating the score of question 2 (which assesses the presence and intensity of LBP). To evaluate the nutritional status of the subjects, BMI and data regarding the preoperative serum levels of total protein (g/dL), albumin (g/dL) and hemoglobin (g/dL) and the total lymphocyte count (10^2^/μL) were collected via chart review.

### Statistical analysis

All data are presented as means and standard deviations. The Shapiro–Wilk test was used to assess the normality of the distribution of the data. Differences in continuous variables between two groups were examined using the Student’s t-test for parametric data and the Mann–Whitney U test for nonparametric data. Multivariate logistic regression analyses were performed to determine the factors significantly associated with LBP. Spearman’s correlation analysis was used to evaluate the correlations of measures with the degree of LBP (5-point NRS score), and Pearson’s correlation analysis was used to evaluate the correlations of measures with ATMS, and the GLFS-24 score. Cohen d effect sizes were also calculated to determine the magnitude of the group differences under the two conditions; they were considered trivial (<0.20), small (0.20–0.50), moderate (0.50–0.80), or large (≥0.80) [33]. All statistical analyses were performed using SPSS version 25 (IBM Corp., Armonk, NY, USA). Analysis items with P value <0.05 were considered statistically significant.

## Results

None of the subjects involved in the present study reported pain during the muscle strength measurements, including ATMS measurements using the device. According to the univariate analysis, there were no differences between the LBP and non-LBP groups regarding age, BMI, grip power, and serum levels of total protein and total lymphocyte count. However, the ATMS, KEMS, and serum albumin and hemoglobin levels were significantly lower in the LBP group than in the non-LBP group. GLFS-24 scores were significantly higher in the LBP group (Table 2). They corresponded to effect sizes of d = 0.91, d = 0.60, d = 0.98, d = 0.61, and d = 0.59, which are considered large or moderate effect sizes. The multivariate logistic regression analysis showed that ATMS (odds ratio, 0.82; P = 0.03) and GLFS-24 scores (odds ratio, 1.04; P <0.01) were independently associated with LBP (Table 3).

**Table 2:** Univariate analysis of differences between the LBP and non-LBP groups.

Measure	LBP group	Non-LBP group	*p* value
**No. of patients**	36	65	
**Physical measurements**			
**Age (years), mean ± SD**	70.9 ± 6.9	69.0 ± 6.0	0.17
**BMI (kg/cm**^**2**^**), mean ± SD**	23.6 ± 3.6	23.7 ± 4.0	0.92
**Grip power (kg), mean ± SD**	19.7 ± 5.8	21.2 ± 5.6	0.20
**ATMS (kPa), mean ± SD**	4.0 ± 2.4	6.0 ± 3.4	<0.01
**KEMS (N/kg), mean ± SD**	3.2 ± 1.2	3.8 ± 1.3	0.03
**GLFS-24 Scores, mean ± SD**	52.1 ± 19.9	35.1 ± 19.9	<0.01
**Blood biomarkers associated with risk of malnutrition**			
**Total protein (g/dL), mean ± SD**	7.2 ± 0.4	7.1 ± 0.4	0.57
**Albumin (g/dL), mean ± SD**	4.1 ± 0.3	4.3 ± 0.2	0.02
**Hemoglobin (g/dL), mean ± SD**	12.6 ± 1.1	13.1 ± 1.1	0.02
**Total lymphocyte (10**^**2**^**/μL), mean ± SD**	16.8 ± 5.5	17.7 ± 6.5	0.52

ATMS, abdominal trunk muscle strength; BMI, body mass index

GLFS-24, 24-Question Geriatric Locomotive Function Scale

KEMS, knee extensor muscle strength; LBP, low back pain; SD, standard deviation

**Table 3:** Multivariate logistic regression analysis of the factors associated with LBP.

Factors	OR	95% CI	*p* value
**ATMS (kPa)**	0.823	0.688–0.985	0.03
**KEMS (N/kg)**	0.883	0.592–1.317	0.54
**GLFS-24 Scores**	1.035	1.010–1.062	<0.01
**Albumin (g/dL)**	0.227	0.026–1.944	0.18
**Hemoglobin (g/dL)**	0.871	0.539–1.408	0.57

ATMS, abdominal trunk muscle strength; CI, confidence interval; GLFS-24, 24-Question Geriatric Locomotive Function Scale; LBP, low back pain; OR, odds ratio

Table 4 shows the correlations of measures with the degree of LBP, ATMS, and GLFS-24 scores. The intensity of LBP had a mildly negative correlation with ATMS (simple regression coefficients: b = -0.26; r^2^ = 0.07; P = 0.01) and KEMS (b = -0.23; r^2^ = 0.05; P = 0.02), and a mildly positive correlation with the GLFS-24 score (b = 0.39; r^2^ = 0.15; P < 0.01). They corresponded to effect sizes of d = 0.66, d = 0.55, and d = 0.95, which are considered large or moderate effect sizes. However, there was no correlation between the intensity of LBP and the other items evaluated. The serum hemoglobin level showed a mildly negative correlation with the GLFS-24 score (b = -0.35; r^2^ = 0.126; P < 0.01) and large effect size (d = 0.95) (Table 4).

**Table 4:** Correlation with the degrees of LBP, abdominal trunk muscle strength, and locomotive syndrome among all 101 participants.

	5-point NRS score for LBP		ATMS (kPa)		GLFS-24 score	
	R value	P value	R value	P value	R value	P value
**Age (years)**	0.13	0.21	-0.14	0.16	0.15	0.15
**BMI (kg/cm**^**2**^**)**	0.14	0.16	0.02	0.82	-0.03	0.75
**Grip power (kg)**	-0.09	0.37	0.38	<0.01	-0.23	0.02
**ATMS (kPa)**	-0.23	0.01	—	—	-0.19	0.06
**KEMS (N/kg)**	-0.21	0.02	0.41	<0.01	-0.24	0.02
**GLFS-24 Scores**	0.35	<0.01	-0.19	0.06	—	—
**Total protein (g/dL)**	0.14	0.15	-0.11	0.29	0.03	0.79
**Albumin (g/dL)**	-0.06	0.57	0.11	0.27	-0.17	0.09
**Hemoglobin (g/dL)**	-0.16	0.12	0.14	0.17	-0.35	<0.01
**Total lymphocyte (10**^**3**^**/μL)**	-0.01	0.91	0.05	0.61	0.04	0.70

ATMS, abdominal trunk muscle strength; BMI, body mass index; GLFS-24, 24-Question Geriatric Locomotive Function Scale; KEMS, knee extensor muscle strength; LBP, low back pain; NRS, numerical rating scale

## Discussion

The current study examined whether muscle strength, including ATMS measured using an innovative device, mobility dysfunction assessed using the LS assessment (the GLFS-24 score), and nutrition status assessed using preoperative non-acute laboratory data and BMI were associated with LBP among older women. Our results demonstrated that the ATMS and KEMS of the subjects with considerable LBP (NRS score ≥2) were significantly lower than those of subjects without LBP. The GLFS-24 scores were higher among subjects with considerable LBP than among subjects without LBP. Multivariate analyses revealed that low ATMS and advanced LS were associated with LBP. However, grip power and KEMS were not associated with LBP. According to the univariate analyses, subjects with considerable LBP had lower serum albumin and hemoglobin levels compared with subjects without LBP. Furthermore, the serum hemoglobin level was correlated with mobility function.

Previous studies have reported that patients with LBP have weaker trunk muscles than asymptomatic individuals [6, 7, 34, 35]. However, most of the strength measurements used during these studies (including sit-ups [34], hyperextension of the trunk [35], and isometric and isokinetic tests using the Cybex [6] and Biodex [7] Systems) imposed strain or stress on the lumbar spine that could induce LBP. LBP induced during strength measurements can decrease the muscle strength to be measured. Recently, in the literature, lumbar stabilization exercises, including the plank, side bridge, and pelvic tilt, have been recommended to improve LBP and physical function [36, 37]. However, many older patients with LBP, other locomotive organ disorders, and/or fragility are unable to perform these exercises because they induce pain in the extremities and spine with significant strain or stress. During strength measurements or exercises using the device, subjects are able to contract the abdominal trunk muscles while sitting without moving the trunk. This isometric muscle contraction does not strain the spine or extremities. Therefore, it may be feasible for older patients to use the device. We previously demonstrated that exercises performed using the device significantly increased strength and activated the abdominals, diaphragm, and pelvic floor muscles [20]. ATMS, as measured by the device, is generated by the contraction of these abdominal core muscles to increase the intra-abdominal pressure and lumbar stability. The results of the present study indicated that ATMS weakness measured using the device was associated with LBP among older women. A pilot study involving older patients with chronic LBP and deteriorated physical ability demonstrated that strengthening exercises performed using the device effectively improved chronic LBP and mobility function [30].

Previous studies have reported that chronic LBP was associated with decreased mobility function in older populations [29, 38]. The results of the current study showed that severe LS was associated with LBP among older women who were scheduled to undergo surgery because of degenerative lower extremity diseases. Because of advanced diseases of the lower extremities requiring surgery, participants involved in the present study had advanced LS and higher LS test scores compared with the older general population [39]. Even with the advanced LS condition of the lower extremity, the presence of LBP was significantly related to the severity of the decreased mobility function assessed using the LS test (the GLFS-24 score eliminated the score for LBP from the GLFS-25 score). Chronic pain including LBP in the older population reduces mobility function [40]. Therefore, exercise and physical activity are recommended to decrease LBP and disability [10, 11, 18, 40]. However, not all treatments options normally indicated for the young population can be applied to the older population, because they have muscle weakness, osteoporosis, and other musculoskeletal pain and dysfunction in the extremities [10–13, 18]. Adherence and motivation are also important for exercise therapy for older patients [9]. Further studies and efforts are required to resolve these problems.

The present study examined the association between the presence of LBP and nutritional status. Most of the nutritional assessment tools included BMI as a criterion for defining malnutrition. In the present study, we were able to collect the non-acute phase laboratory data of all subjects, including data regarding serum levels of total protein, albumin, and hemoglobin, total lymphocyte count; and BMI, because the laboratory data were obtained routinely under stable preoperative and non-acute conditions, which made patients eligible for surgery for degenerative musculoskeletal diseases. Furthermore, patients with inflammatory diseases were excluded from the analyses performed during the study. These biomarkers were also proven to be associated with the risk of malnutrition among older adults [41, 42]. In the present study, serum albumin and hemoglobin levels were significantly lower in the LBP group than in the non-LBP group, but they were not associated with LBP in the multivariate analysis. Several inflammatory biomarkers have been reported to be associated with acute or chronic LBP [43, 44]. However, no studies have reported an association between hypoalbuminemia or anemia and LBP. Interestingly, the serum hemoglobin level was negatively correlated with decreased mobility function (GLFS-24 score). A systematic review and meta-analysis concluded that BMI, hemoglobin, and total cholesterol were useful biomarkers of malnutrition in older adults [41]. The malnutrition risk that was represented by lower serum hemoglobin levels in older adults was related to decreased mobility function. A systematic review highlighted that physical activity can decrease chronic LBP and improve disability in older patients [10]. The relationship of malnutrition with decreased mobility may also affect the presence of LBP in older adults. However, future studies involving a larger cohort of older individuals and detailed nutritional risk screening should aim to identify the malnutrition risk factors that can be associated with LBP and muscle strength as well as decreased mobility function.

The current study had several limitations, including the relatively small sample size and the inclusion of many participants with locomotive organ disorders of the lower extremities, which may have impacted the results. The absence of a correlation between ATMS and decreased mobility function (GLFS-24 scores) may have occurred because of the presence of other musculoskeletal diseases. In this study, the malnutrition risk was evaluated only using blood biomarkers and BMI. The evaluation did not include the preoperative dietary status. The cause of LBP among the subjects might be multifactorial; however, it was not evaluated. Both acute and chronic LBP were assessed by question 2 of the GLFS-25 during the present study. Back muscle strength, which has been reported to be associated with LBP and physical function among older people [45, 46], was not measured in the present study. Despite its limitations, the present study showed that older patients with LBP had lower ATMS and decreased mobility function than those without LBP. Our device is a viable option for measuring ATMS as a factor associated with LBP.

## Conclusion

Abdominal trunk muscle weakness and advanced LS were associated with LBP in older women. Further studies are needed to validate the association of malnutrition with LBP and muscle strength as well as decreased mobility function.

## Supporting information

S1 File (DOCX)
