# Supplementary material for: Association of low back pain with muscle weakness, decreased mobility function, and malnutrition in older women: A cross-sectional study
Source: PLoS One. 2021 Jan 25;16(1):e0245879. doi: 10.1371/journal.pone.0245879 (PMC7833166; doi:10.1371/journal.pone.0245879)
Supplement: S1 File — (DOCX) [file pone.0245879.s001.docx]

**S1 Data**

Data of the 36 patients in the LBP group

| No | Age  (years) | BMI  (kg/cm^2^) | GP  (kg) | ATMS  (kPa) | KEMS  (N/kg) | GLFS  -24 | TP  (g/dL) | Alb  (g/dL) | Hb  (g/dL) | TLC  (10^2^/µL) | NRS-LBP |
| --- | --- | --- | --- | --- | --- | --- | --- | --- | --- | --- | --- |
| 1 | 60 | 23.1 | 34 | 1.2 | 2.5 | 55 | 7.2 | 4.4 | 11.7 | 12.6 | 2 |
| 2 | 61 | 25 | 21 | 4.1 | 2.6 | 8 | 6.6 | 4 | 14.9 | 6.4 | 3 |
| 3 | 62 | 21.5 | 24 | 2.1 | 3.91 | 42 | 7.7 | 3.9 | 12.2 | 21.8 | 2 |
| 4 | 62 | 29.6 | 17 | 4.2 | 2.56 | 33 | 7.8 | 4.2 | 14.5 | 31.3 | 3 |
| 5 | 62 | 25.8 | 27.5 | 5.6 | 4.85 | 45 | 7.3 | 4.7 | 13.8 | 15.5 | 2 |
| 6 | 64 | 22.7 | 31 | 5.7 | 4.77 | 70 | 7.5 | 4.2 | 13 | 18.1 | 3 |
| 7 | 64 | 27.2 | 13.5 | 5.7 | 0.9 | 49 | 7.5 | 4.3 | 13 | 10.9 | 3 |
| 8 | 65 | 18 | 21 | 3.7 | 2.93 | 26 | 7 | 4.3 | 12.6 | 14.7 | 3 |
| 9 | 66 | 25.4 | 10 | 1.6 | 1.2 | 42 | 7.2 | 4.4 | 13.7 | 20.2 | 3 |
| 10 | 67 | 17.8 | 23 | 9.1 | 4.9 | 46 | 6.8 | 4.1 | 12.1 | 16.5 | 3 |
| 11 | 67 | 22.3 | 22 | 3.2 | 1.81 | 64 | 7.3 | 4.4 | 13 | 19.8 | 3 |
| 12 | 68 | 19.2 | 18 | 5.2 | 2.8 | 82 | 7.1 | 3.8 | 12 | 12.5 | 2 |
| 13 | 68 | 22 | 21 | 8.9 | 5.22 | 47 | 7 | 3.8 | 13 | 15.9 | 2 |
| 14 | 68 | 23.6 | 24.5 | 5.4 | 5.9 | 7 | 6.6 | 4 | 12.3 | 18.9 | 2 |
| 15 | 68 | 17.5 | 20.5 | 2.7 | 3.5 | 58 | 6.8 | 4.5 | 11.5 | 15.5 | 2 |
| 16 | 68 | 27.6 | 21 | 2.6 | 3 | 57 | 7.4 | 4.6 | 13.3 | 15.5 | 3 |
| 17 | 68 | 30.1 | 20 | 7 | 3.5 | 38 | 7.6 | 4.7 | 14 | 12.3 | 2 |
| 18 | 69 | 21.4 | 18.5 | 4.6 | 3.9 | 50 | 7.3 | 4.1 | 12.2 | 14.5 | 3 |
| 19 | 69 | 18.2 | 20 | 2.5 | 3.1 | 56 | 6.6 | 4.2 | 12.7 | 9.5 | 3 |
| 20 | 70 | 27.6 | 27.5 | 5.1 | 1.6 | 58 | 7.3 | 4.1 | 14.7 | 25.8 | 3 |
| 21 | 71 | 25.6 | 10.5 | 2.5 | 1.84 | 90 | 7.3 | 4.3 | 12 | 16.7 | 3 |
| 22 | 72 | 21 | 11 | 1.4 | 2.5 | 21 | 6.6 | 3.7 | 11.5 | 12.3 | 2 |
| 23 | 72 | 21.6 | 13.5 | 2.4 | 2.46 | 68 | 6.9 | 3.8 | 11.8 | 20.9 | 2 |
| 24 | 72 | 26.4 | 23 | 5.3 | 1.73 | 82 | 7.4 | 4.2 | 10.7 | 13.7 | 3 |
| 25 | 74 | 28.8 | 13 | 2.9 | 2.3 | 72 | 6.9 | 4.2 | 13.1 | 25.6 | 3 |
| 26 | 74 | 22.7 | 20 | 3.2 | 2.9 | 59 | 6.7 | 4.3 | 11.3 | 14.7 | 2 |
| 27 | 75 | 26.5 | 20 | 10.3 | 3.72 | 59 | 7 | 3.8 | 12.4 | 28.7 | 3 |
| 28 | 75 | 19.6 | 19.5 | 1.3 | 3.6 | 32 | 8.5 | 4 | 12.7 | 15.3 | 2 |
| 29 | 78 | 29.6 | 24 | 5.3 | 3.4 | 74 | 7.5 | 4.1 | 13.3 | 13.9 | 3 |
| 30 | 78 | 26.1 | 20 | 4.1 | 4 | 84 | 7.2 | 4.1 | 12.8 | 14.2 | 4 |
| 31 | 79 | 27.1 | 13 | 0.4 | 2.3 | 50 | 8 | 4.3 | 12.1 | 17.9 | 3 |
| 32 | 80 | 20.8 | 25 | 7 | 4.8 | 57 | 6.5 | 4 | 12.6 | 10.3 | 2 |
| 33 | 82 | 24 | 21 | 2.7 | 4.69 | 63 | 6.9 | 3.9 | 10.8 | 15.8 | 2 |
| 34 | 83 | 24 | 15 | 3.4 | 2.58 | 53 | 7.6 | 4 | 13.7 | 24 | 3 |
| 35 | 84 | 19 | 8.5 | 0.4 | 4.6 | 52 | 7.6 | 3.9 | 10.8 | 22 | 2 |
| 36 | 86 | 21.8 | 17 | 1.2 | 1.8 | 26 | 6.6 | 4 | 11.9 | 12.1 | 2 |

Alb, Albumin; ATMS, abdominal trunk muscle strength; BMI, body mass index; GLFS-24, 24-Question Geriatric Locomotive Function Scale; GP, grip power; Hb, Hemoglobin, KEMS, knee extensor muscle strength; LBP, low back pain; NRS-LBP, numerical rating scale for low back pain, TLC, total lymphocyte, TP, total protein

Data of the 65 patients in the non-LBP group

| No | Age  (years) | BMI  (kg/cm^2^) | GP  (kg) | ATMS  (kPa) | KEMS  (N/kg) | GLFS  -24 | TP  (g/dL) | Alb  (g/dL) | Hb  (g/dL) | TLC  (10^2^/µL) | NRS-LBP |
| --- | --- | --- | --- | --- | --- | --- | --- | --- | --- | --- | --- |
| 1 | 60 | 26.6 | 31.5 | 6.9 | 5.5 | 45 | 7 | 4 | 12.8 | 24.2 | 1 |
| 2 | 60 | 20 | 10 | 9.1 | 2.72 | 23 | 6.9 | 4.1 | 13.7 | 24 | 0 |
| 3 | 60 | 21.8 | 21 | 11.8 | 3.2 | 60 | 7.5 | 4.3 | 12.2 | 13.9 | 0 |
| 4 | 61 | 22.6 | 21.5 | 5.6 | 1.8 | 56 | 6.5 | 3.8 | 11.7 | 17.8 | 0 |
| 5 | 61 | 22.4 | 26 | 10.2 | 5.1 | 70 | 6.6 | 4.1 | 12.2 | 13.3 | 1 |
| 6 | 61 | 18.4 | 24.5 | 0.9 | 1.4 | 26 | 7.7 | 4.2 | 12.3 | 7.3 | 1 |
| 7 | 61 | 24.8 | 20 | 5.3 | 4.3 | 8 | 6.9 | 4.3 | 13.1 | 8.2 | 1 |
| 8 | 61 | 21.8 | 39.5 | 14.8 | 6.3 | 10 | 7.3 | 4.4 | 12.6 | 22.2 | 0 |
| 9 | 62 | 18.9 | 24.5 | 4.1 | 4.6 | 45 | 6.1 | 3.7 | 12.1 | 10.4 | 0 |
| 10 | 62 | 21 | 19.5 | 1.5 | 3.4 | 42 | 6.7 | 4.2 | 14.7 | 20.9 | 1 |
| 11 | 63 | 23.6 | 22 | 4.5 | 1.89 | 9 | 6.6 | 4.1 | 13.3 | 9.8 | 0 |
| 12 | 63 | 26.1 | 28.5 | 11.6 | 5.6 | 14 | 6.8 | 4.1 | 15.1 | 10.9 | 0 |
| 13 | 63 | 22.2 | 17.5 | 4.9 | 3.1 | 41 | 7.2 | 4.1 | 12.5 | 14.7 | 0 |
| 14 | 63 | 20.8 | 22.5 | 6.5 | 5.8 | 32 | 6.7 | 4.2 | 14.1 | 19.6 | 1 |
| 15 | 63 | 23.4 | 24 | 4.9 | 3.8 | 41 | 7.1 | 4.2 | 14.1 | 31.9 | 0 |
| 16 | 64 | 17 | 18 | 5.2 | 5.7 | 72 | 7.1 | 4.3 | 11.9 | 12.5 | 0 |
| 17 | 64 | 32.5 | 21 | 2.8 | 1.8 | 28 | 7.1 | 4.3 | 14.6 | 38.4 | 1 |
| 18 | 65 | 30.3 | 21.5 | 6.2 | 3.2 | 47 | 7.3 | 4.1 | 12.9 | 24 | 0 |
| 19 | 65 | 21.3 | 29.5 | 8.4 | 5.2 | 44 | 7.2 | 4.4 | 14.1 | 20.6 | 1 |
| 20 | 65 | 26.3 | 22 | 7 | 3.1 | 11 | 7.9 | 4.5 | 13 | 23.2 | 1 |
| 21 | 66 | 22.8 | 18.5 | 5.7 | 3.74 | 71 | 7.6 | 4 | 11.9 | 29.1 | 1 |
| 22 | 66 | 21.8 | 24 | 1.3 | 5.68 | 32 | 7.5 | 4.7 | 12.8 | 15.7 | 1 |
| 23 | 66 | 21.9 | 21 | 6.5 | 4.1 | 50 | 7.6 | 4.8 | 15.2 | 18 | 1 |
| 24 | 67 | 29.8 | 23 | 6.9 | 4.4 | 52 | 7 | 4 | 13.2 | 24.5 | 0 |
| 25 | 67 | 23.5 | 23 | 5.1 | 4.3 | 18 | 6.8 | 4.2 | 13.1 | 14.1 | 1 |
| 26 | 67 | 16.7 | 17 | 2 | 5.1 | 58 | 6.6 | 4.2 | 11.7 | 20.1 | 0 |
| 27 | 67 | 26.7 | 23 | 3.3 | 2 | 24 | 7.4 | 4.2 | 14.5 | 26.2 | 0 |
| 28 | 67 | 23.7 | 22 | 14.1 | 5.2 | 17 | 7.5 | 4.3 | 14.1 | 7.1 | 1 |
| 29 | 67 | 26.4 | 20 | 6.6 | 3.2 | 29 | 7.8 | 4.6 | 13.3 | 29 | 1 |
| 30 | 68 | 22.6 | 20.5 | 6 | 5.01 | 22 | 7 | 4.1 | 13 | 21.6 | 0 |
| 31 | 68 | 23 | 24 | 2.4 | 3.1 | 8 | 7.1 | 4.2 | 12.9 | 9.2 | 0 |
| 32 | 68 | 25.6 | 11.5 | 2.5 | 2.58 | 30 | 6.8 | 4.2 | 13.8 | 12.5 | 0 |
| 33 | 69 | 25.3 | 26 | 10.3 | 3.7 | 37 | 6.3 | 4.1 | 13 | 16.5 | 1 |
| 34 | 69 | 25.7 | 24 | 7.9 | 4.6 | 8 | 7.3 | 4.3 | 13.8 | 17.1 | 1 |
| 35 | 70 | 17.8 | 13.5 | 1.9 | 1.34 | 15 | 7.2 | 4 | 13.1 | 17.4 | 0 |
| 36 | 70 | 21 | 25.5 | 5.3 | 5.4 | 16 | 6.9 | 4.1 | 13.6 | 17 | 0 |
| 37 | 70 | 22 | 22 | 2.2 | 3.5 | 19 | 7.4 | 4.2 | 12.1 | 15 | 1 |
| 38 | 70 | 23.8 | 29 | 7.4 | 3.9 | 10 | 7.3 | 4.2 | 13 | 18.2 | 0 |
| 39 | 70 | 23 | 28 | 10.5 | 4.1 | 36 | 6.7 | 4.3 | 13.8 | 11.9 | 0 |
| 40 | 70 | 31.6 | 8 | 6.3 | 2.8 | 40 | 7.4 | 4.4 | 12.5 | 23.8 | 1 |
| 41 | 70 | 19.2 | 15.5 | 1.4 | 3 | 63 | 7.3 | 4.6 | 12.8 | 11.6 | 0 |
| 42 | 70 | 23.7 | 29 | 7.4 | 4.5 | 20 | 7.9 | 4.7 | 13.5 | 12.7 | 1 |
| 43 | 70 | 25.5 | 26 | 11.2 | 4.8 | 32 | 7.5 | 4.7 | 13.6 | 13.3 | 1 |
| 44 | 71 | 29.4 | 24 | 4.3 | 3.59 | 27 | 7.8 | 4.2 | 11.9 | 25.6 | 0 |
| 45 | 71 | 23.4 | 12 | 7.6 | 0.82 | 61 | 6.8 | 4.3 | 13.7 | 17.3 | 1 |
| 46 | 71 | 23.1 | 17 | 4.7 | 5.24 | 13 | 7.3 | 4.5 | 14.2 | 9.8 | 0 |
| 47 | 73 | 36.7 | 16 | 1.7 | 0.9 | 63 | 7.2 | 4.1 | 13.2 | 18.3 | 1 |
| 48 | 73 | 29.3 | 8 | 3.6 | 3.1 | 49 | 7.2 | 4.2 | 12.8 | 13.7 | 0 |
| 49 | 73 | 19.6 | 20.5 | 7.6 | 6.02 | 10 | 7 | 4.4 | 13.5 | 18 | 1 |
| 50 | 74 | 31.4 | 12.5 | 1.9 | 2.71 | 43 | 6.9 | 3.9 | 13.7 | 11.4 | 1 |
| 51 | 74 | 21.3 | 23 | 9.4 | 3.2 | 12 | 6.6 | 4.2 | 11.1 | 12 | 1 |
| 52 | 75 | 17.5 | 15.5 | 3.7 | 2.1 | 75 | 6.8 | 3.8 | 12.3 | 13.8 | 0 |
| 53 | 75 | 20 | 20 | 0.9 | 3.9 | 73 | 7.4 | 4.4 | 11.3 | 14.5 | 1 |
| 54 | 75 | 30.6 | 22 | 3.7 | 3.5 | 19 | 7.3 | 4.4 | 14.7 | 20.7 | 1 |
| 55 | 75 | 25.5 | 19.5 | 3.5 | 4.33 | 38 | 7.8 | 4.5 | 13.6 | 17.6 | 1 |
| 56 | 76 | 20.1 | 19 | 5.8 | 3.84 | 65 | 7 | 4.2 | 13.7 | 20.2 | 0 |
| 57 | 76 | 24 | 16 | 9.9 | 3.6 | 15 | 7.3 | 4.6 | 15.9 | 33.6 | 0 |
| 58 | 77 | 26.8 | 26 | 11.1 | 4.6 | 31 | 6.8 | 4.2 | 12.1 | 14.9 | 1 |
| 59 | 77 | 18.9 | 19 | 12.7 | 3.5 | 58 | 7.2 | 4.2 | 9.4 | 21.3 | 0 |
| 60 | 77 | 24 | 19 | 8.7 | 4.2 | 26 | 7.4 | 4.5 | 12.1 | 22.7 | 1 |
| 61 | 78 | 20.9 | 26 | 4.3 | 5.1 | 29 | 7.2 | 4.4 | 13 | 18.2 | 1 |
| 62 | 81 | 23.4 | 22 | 5 | 3.5 | 46 | 7.1 | 4.2 | 11.8 | 12.7 | 1 |
| 63 | 81 | 23.6 | 26.5 | 2.2 | 4.74 | 10 | 7 | 4.4 | 13.4 | 16.2 | 0 |
| 64 | 82 | 23 | 17 | 4 | 2.7 | 65 | 7.8 | 4.5 | 15 | 14.5 | 1 |
| 65 | 84 | 23.2 | 20 | 9.7 | 2.9 | 25 | 7.1 | 4.2 | 15 | 12.1 | 0 |

Alb, Albumin; ATMS, abdominal trunk muscle strength; BMI, body mass index; GLFS-24, 24-Question Geriatric Locomotive Function Scale; GP, grip power; Hb, Hemoglobin, KEMS, knee extensor muscle strength; LBP, low back pain; NRS-LBP, numerical rating scale for low back pain, TLC, total lymphocyte, TP, total protein
